# Surgical extraction of a metal ring embedded in the penis for five years: a case report

**DOI:** 10.3389/fsurg.2026.1807432

**Published:** 2026-04-15

**Authors:** Chaodong Shen, Mengqi Shi, Zhirong Zhu

**Affiliations:** Department of Urology, Shaoxing People's Hospital, Shaoxing Hospital of Zhejiang University, Shaoxing, Zhejiang, China

**Keywords:** case report, chronic penile strangulation, foreign body, subcutaneous, surgical treatment

## Abstract

**Introduction:**

Penile strangulation represents an uncommon urological emergency that has been increasingly reported in recent literature. However, chronic penile strangulation resulting from subcutaneous embedding of foreign objects is even more rare.

**Case presentation:**

A male patient presented to the urology outpatient clinic with progressive swelling at the penile base and purulent discharge from the urethral meatus. Clinical evaluation revealed a metal ring that had been placed at the penile base for five years and had gradually become embedded beneath the skin, rendering it invisible to the naked eye. During surgery, an annular metal object was exposed following an incision of the penile skin and successfully removed without urethral injury. At one-month postoperative follow-up, proper wound healing and normal urinary function were observed.

**Conclusion:**

This report describes an exceptionally rare case of chronic penile strangulation. Successful surgical removal of the embedded metal ring was achieved with minimal complications.

## Introduction

Penile strangulation caused by foreign objects is an uncommon urological emergency that can result in progressive edema, local ischemia, and tissue necrosis, necessitating immediate diagnosis and treatment. Previous research has documented a range of commonly encountered foreign objects, including metal rings, hair tourniquets, washers, and rubber bands (1). In contrast, cases of chronic penile strangulation caused by subcutaneous embedding of similar foreign bodies are exceedingly rare (2). This report presents an uncommon case of chronic penile strangulation caused by a metal ring retained for five years.

## Case presentation

A 37-year-old male presented to the outpatient clinic in March 2025 with a one-month history of swelling and pain at the penile base, accompanied by purulent discharge from the urethral meatus. Five years earlier, the patient had placed a metal ring around the base of his penis and, for reasons unknown, failed to remove it. Over time, the ring progressively eroded the overlying skin and became embedded within the subcutaneous tissue. Throughout this period, no significant signs of ischemia were reported. Urinary function remained normal, with no evident effect on his daily activities; nonetheless, episodes of erectile dysfunction were reported. The patient developed pain at the base of the penis, accompanied by purulent discharge from the urethral opening one month ago. Due to the involvement of a private area and the mild nature of the pain, the patient did not seek medical attention. Medical attention was sought only after swelling developed at the penile base and progressively worsened.

**Figure 1:**
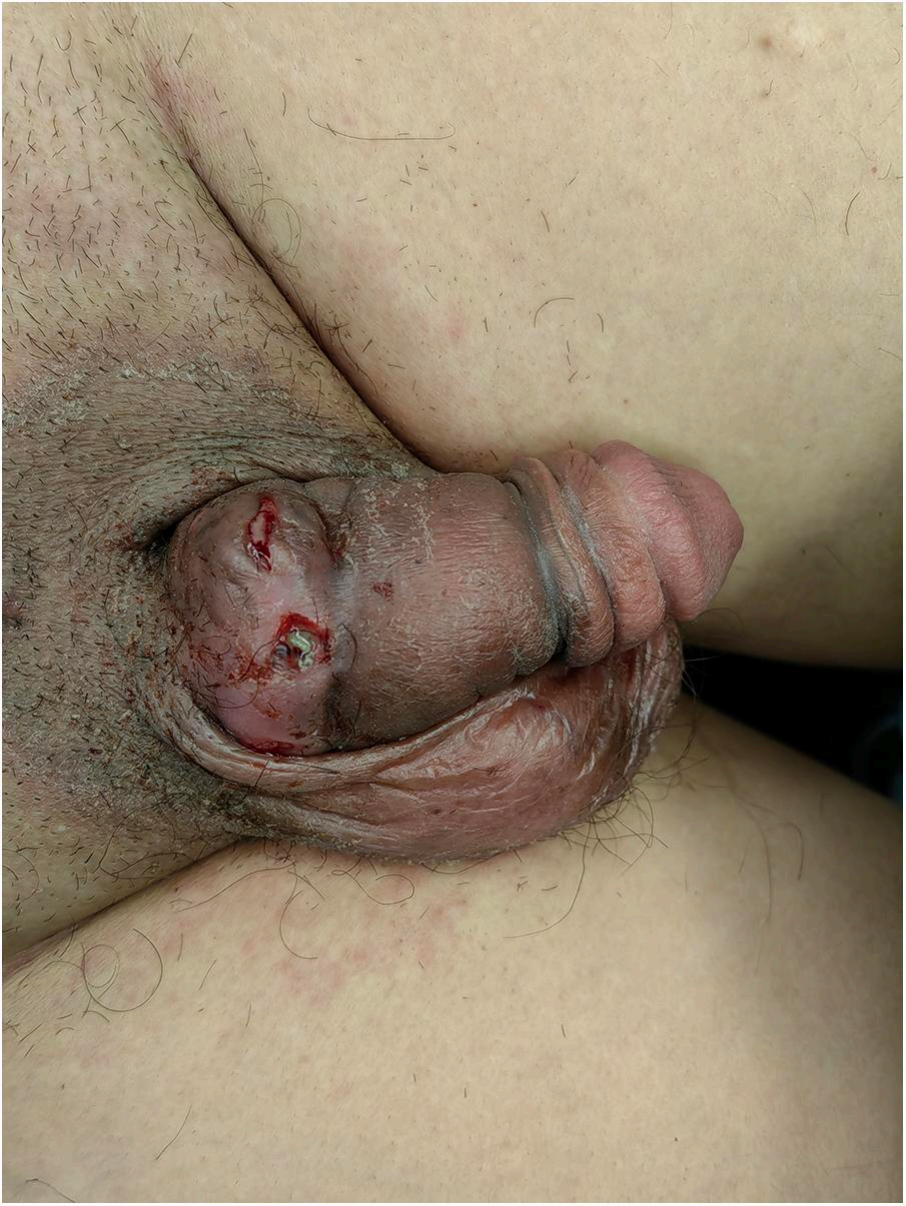
Preoperative view of penile strangulation illustrating circumferential swelling at the base of the penis, multiple areas of skin ulceration, and a small amount of purulent discharge.

**Figure 2:**
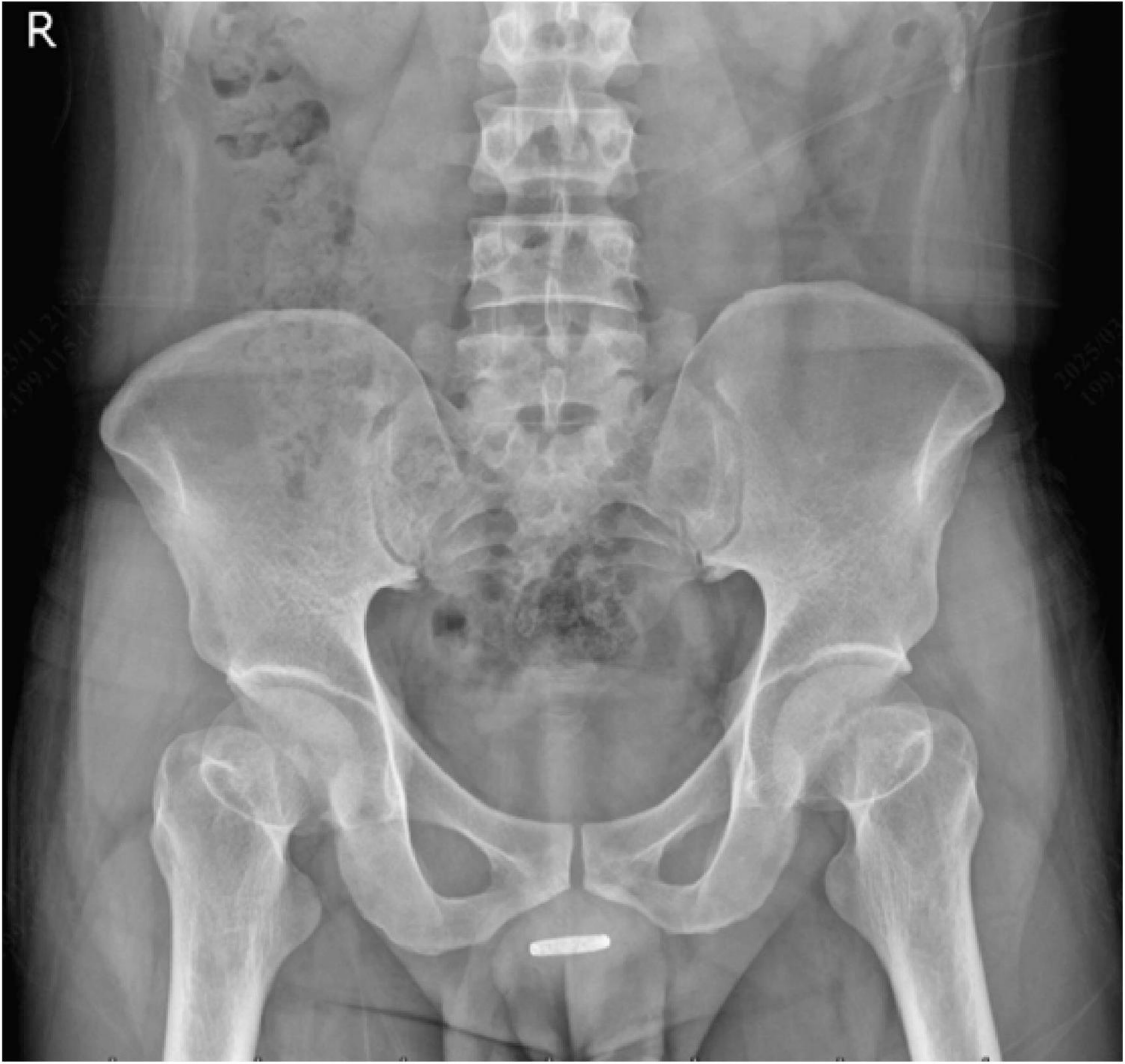
Plain x-ray examination confirms the presence of an embedded metal ring at the penile base.

Physical examination revealed circumferential swelling at the penile base with multiple areas of skin ulceration and minimal purulent discharge. No foreign object was visible on inspection, and palpation did not detect any obvious foreign object. Penile sensation was intact (Figure 1). A plain x-ray examination confirmed the presence of a metal ring at the base of the penis (Figure 2). The patient maintained normothermia, while urinalysis and urine culture exhibited no significant signs of urinary tract infection. We performed screening for common sexually transmitted infections, including but not limited to Neisseria gonorrhoeae and Chlamydia trachomatis, and all results were negative. Assessment of erectile function via the IIEF-5 scale showed mild erectile dysfunction. Urodynamic studies showed satisfactory uroflowmetry parameters, indicating unobstructed voiding and no evidence of urethral stricture or other complications. The patient complete a psychological assessment scale and scored 3 points. The institutional threshold for psychiatric consultation is >11 points, so the patient did not meet the referral criteria. Nevertheless, we discussed the option of a psychiatric consultation about the patient. The patient was informed but strongly refused.

**Figure 3:**
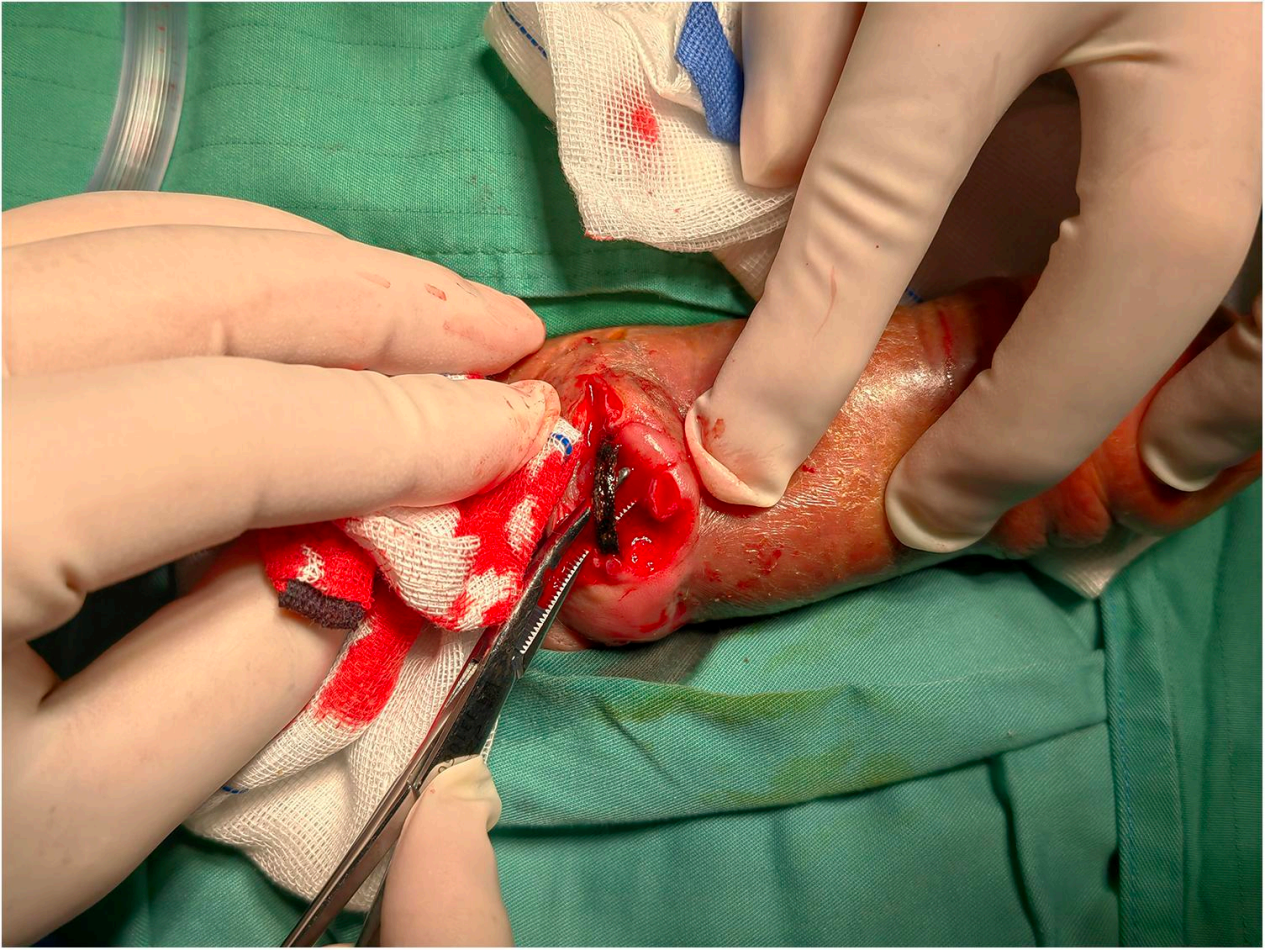
Intraoperative visualization of the metal ring.

**Figure 4:**
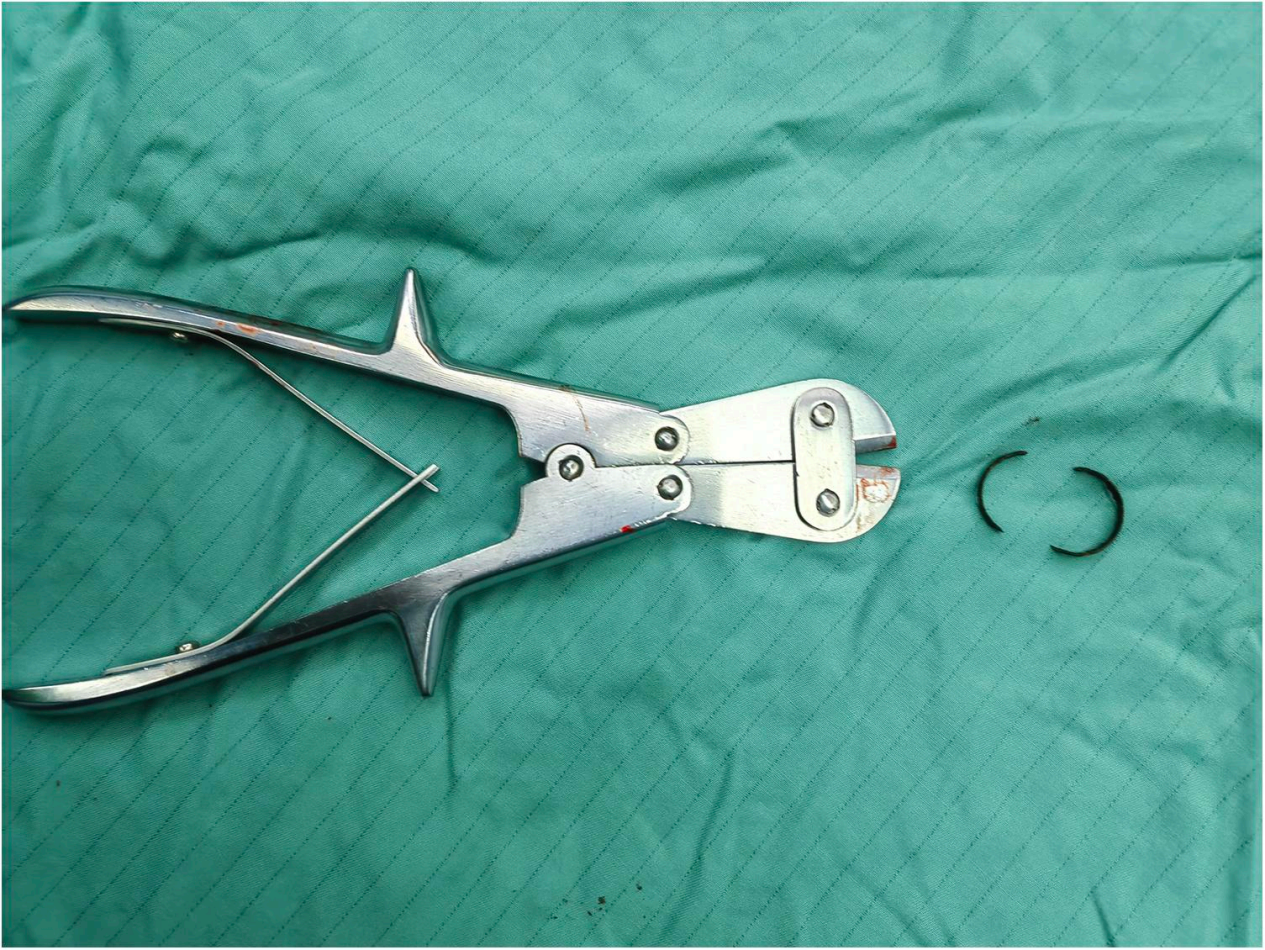
Fragmentation and removal of the ring using wire-cutting pliers and a hemostatic clamp.

Fire department personnel were contacted in advance to prepare the necessary cutting equipment. The patient received general anesthesia after three days of antimicrobial therapy. Following thorough disinfection, a transverse incision was made on the dorsal penile skin at the rupture site. No significant purulent discharge was observed beneath the subcutaneous tissue. Upon downward dissection of the tissue plane, the embedded metal ring was exposed without urethral involvement. While the dorsal neurovascular bundle was preserved, minimal tissue adhesion was noted with the ring conspicuously thin and fragile (Figure 3). The ring was carefully fragmented and extracted using wire-cutting pliers and a hemostatic clamp (Figure 4). The ring integrity was ascertained, and the penile tissue was assessed to ensure no retained fragments remain before wound closure. The diameter of the ring measured 2 cm. A urinary catheter was indwelled and was withdrawn in good condition three days after surgery.

No complications were observed, including voiding difficulty, urethral fistula, or urinary irritation symptoms during the three-month postoperative follow-up period. Additionally, erectile function remained comparable to preoperative status

## Discussion

Penile strangulation represents a severe urological emergency. Tissue edema exacerbates genital constriction, and prolonged compression may induce penile compartment syndrome, initially impairing venous and lymphatic drainage and subsequently compromising arterial inflow, ultimately resulting in ischemic necrosis (3). Prompt intervention is essential to relieve tissue compression and prevent irreversible complications. Gauthier reported the first case of penile strangulation in 1755 (4). In 1991, Bhat et al. propounded a five-grade classification system for penile strangulation injuries, stratifying cases based on the severity of injury, including skin ulceration, urethral injury, penile sensation impairment, urethral fistula, and gangrene or necrosis of the penis (5). Silberstein subsequently refined this classification system, categorizing injuries into two grades: low-grade (Bhat grades 1–3) injuries, which can be effectively managed with conservative treatment through simple decompression, and high-grade (Bhat grades 4 and 5) injuries that require surgical intervention due to foreign body removal (6). Generally, accurate clinical assessment and appropriate treatment selection remain critical determinants of clinical outcomes.

Most published case reports describe acute presentations requiring urgent medical attention. Campbell et al. (7) reported a case involving penile strangulation caused by a lotion bottle with a 6-h duration of constriction. Clinical evaluation demonstrated reduced penile sensation and urethral fistula formation, necessitating surgical intervention with urethroplasty after foreign body removal. In contrast, reports on chronic penile strangulation are limited. A case report published in 2003 described penile strangulation after prolonged intermittent use of a rubber-enlarging band for sexual enhancement, with medical presentation occurring after 3 years of symptom progression (1). Physical examination revealed penile lymphedema without scrotal involvement. Conservative management was recommended, as the patient maintained satisfaction with both penile appearance and sexual function. Xiao et al. (2) documented a six-month case of chronic penile strangulation caused by a metal ring at the penile base, resulting in severe distal lymphatic edema precluding direct removal. The metal ring was successfully divided using a mini circular saw with thermal injury precautions in operation. Two-month follow-up confirmed complete wound healing without urination dysfunction. Okeke reported a unique pediatric case of a 9-year-old boy with hair-thread tourniquet syndrome affecting the penis for three years (8). Retrograde urethrography demonstrated a tight ring stricture in his urethra, necessitating surgical intervention. The hair thread tourniquet was divided and removed, followed by urethral stricture resection and reconstruction. Postoperative recovery was uneventful, with catheter removal at three weeks and restoration of normal urination function during follow-up. Zhao et al. (9) reported a 73-year-old male who had Penial incarceration with a metallic hoop for three months. The foreign body was lodged at the base of the penis and remained visible to the naked eye. Clinical manifestations were confined to chronic swelling of the penis and voiding dysfunction. Compared to the tools commonly reported in other cases, the surgeon ultimately selected a fretsaw as the primary cutting instrument for the metallic foreign body. Its advantages in terms of operational flexibility and safety are discussed from three perspectives (controllable operation, no risk of spark generation, and avoidance of accidental injury caused by structural damage to the metal). The patient presented with mild preoperative symptoms and no abnormal complications such as voiding difficulties or erectile dysfunction during postoperative follow-up, confirming that chronic low-grade entrapment may not result in irreversible tissue damage.

In the present case, a metal ring was embedded subcutaneously at the base of the penis without external visibility. Despite being retained for five years, the patient exhibited no ischemic skin changes or sensory loss in the penis, with only mild erectile dysfunction. After a thorough clinical evaluation, the injury was classified as low-grade (Bhat Grade 2). This finding suggests that under mild chronic entrapment, patients have the potential to maintain normal voiding function, while erectile function may be mildly affected. Since the material of the metal ring could not be determined, standard surgical instruments were prepared in the operating room, and additional specialized cutting tools were arranged in collaboration with the local fire department to ensure removal. To prevent urethral injury, an indwelling urinary catheter was placed before surgical initiation. As the dissection proceeded, the metal ring was progressively exposed, demonstrating no significant adhesion to surrounding tissues, a phenomenon potentially attributable to the unique anatomical microenvironment of the penis, particularly the loose areolar tissue of Buck's fascia, which provides a natural cleavage plane, limiting firm adhesion. Recurrent expansion and contraction of erectile tissue may further inhibit stable adhesion through continuous micromotion. Additionally, the favorable biocompatibility of the metallic material may have mitigated foreign body reactions, which are typically associated with metal implants. However, limited literature prevents definitive conclusions regarding adhesion patterns in long-term embedded metal rings. Prolonged corrosion rendered the ring remarkably thin and fragile, necessitating removal with wire-cutting pliers. After complete circumferential dissection, the ring was fragmented with the assistance of a hemostatic clamp and extracted piecewise, allowing complete removal with minimal tissue trauma while avoiding penile degloving. Since the urethra exhibited no signs of stenosis or injury, urethral reconstruction was not required. Given the altered consistency of the metal, care should be taken to avoid leaving small fragments in the tissue. If necessary, a plain pelvic radiograph can be performed during the procedure.

Among middle-aged and elderly males, penile constriction practices are usually associated with an attempt to enhance sexual performance or achieve autoerotic stimulation (10). The delayed presentation observed in the patient reflected psychological embarrassment. Postoperative follow-up should extend beyond physical recovery to include assessment of mental health and counselling when necessary.

## Conclusion

Chronic penile strangulation represents an uncommon and challenging clinical condition. When foreign bodies are embedded and not visible, injury classification and functional assessment, comprehensive multidisciplinary collaboration, selection of therapeutic Instruments are essential. Successful removal of a foreign body with minimal procedural complications carries significant prognostic implications for patient recovery.

## Data Availability

The original contributions presented in the study are included in the article/Supplementary Material, further inquiries can be directed to the corresponding author.

## References

[1] Lopes RI Lopes SI Lopes RN. Chronic penile strangulation. Int Braz J Urol. (2003) 29(4):327–9. 10.1590/s1677-55382003000400007 15745556

[2] Xiao Y Xie T Zhu H Yang J. Chronic penile strangulation caused by metal ring: a case report. Asian J Surg. (2023) 46(6):2643–4. 10.1016/j.asjsur.2022.12.148 36635170

[3] Puvvada S Kasaraneni P Gowda RD Mylarappa P Manasa T Dokania K Stepwise approach in the management of penile strangulation and penile preservation: 15-year experience in a tertiary care hospital. Arab J Urol. (2019) 17(4):305–13. 10.1080/2090598x.2019.1647677 31723448 PMC6830290

[4] Noh J Kang TW Heo T Kwon DD Park K Ryu SB. Penile strangulation treated with the modified string method. Urology. (2004) 64(3):591. 10.1016/j.urology.2004.04.058 15351614

[5] Bhat AL Kumar A Mathur SC Gangwal KC. Penile strangulation. Br J Urol. (1991) 68(6):618–21. 10.1111/j.1464-410x.1991.tb15426.x 1773293

[6] Silberstein J Grabowski J Lakin C Goldstein I. Penile constriction devices: case report, review of the literature, and recommendations for extrication. J Sex Med. (2008) 5(7):1747–57. 10.1111/j.1743-6109.2008.00848.x 18507720

[7] Campbell K Terry R Yeung L. Surgical reconstruction and follow-up of penile strangulation injury. Urol Case Rep. (2018) 19:6–8. 10.1016/j.eucr.2018.02.005 29888174 PMC5991326

[8] Okeke LI. Thread embedded into penile tissue over time as an unusual hair thread tourniquet injury to the penis: a case report. J Med Case Rep. (2008) 2:230. 10.1186/1752-1947-2-230 18631393 PMC2491652

[9] Zhao Y Xue XQ Huang HF Xie Y Ji ZG Fan XR. Using a fretsaw in treating chronic penial incarceration: a case report. World J Clin Cases. (2022) 10(2):747–52. 10.12998/wjcc.v10.i2.747 35097103 PMC8771402

[10] Talib RA Canguven O Al Ansari A Shamsodini A. Treatment of penile strangulation by the rotating saw and 4-needle aspiration method: two case reports. Arch Ital Urol Androl. (2014) 86(2):138–9. 10.4081/aiua.2014.2.138 25017597

